# Analysis of Requirements for the Medication Profile to Be Used in Clinical Research: Protocol Feasibility Studies and Patient Recruitment

**DOI:** 10.1155/2015/932935

**Published:** 2015-10-11

**Authors:** Julie M. James, Dipak Kalra, Jane Portlock

**Affiliations:** ^1^UCL CHIME, London WC1E 6BT, UK; ^2^EuroRec, 9000 Gent, Belgium; ^3^University of Portsmouth, Portsmouth PO1 2UP, UK

## Abstract

A “Medication Profile,” the information about the medicines a person is using and has used, is a core part of many electronic health record systems and summaries. However, there is little objective research into the data elements that the profile should contain to support the uses it must serve. With the increasing emphasis on secondary uses of electronic health information, as well as supporting the requirements to support direct to patient care, the Medication Profile should also support the requirements from clinical research. However, there is little, if any, description of these available. This paper describes an analysis of a set of study eligibility criteria that was undertaken to investigate which medication-related data elements would be required to support two clinical research use cases: the parameters to query a patient's Medication Profile to assess their suitability for entry into a trial (patient recruitment) and the parameters to query a set of Medication Profiles in a data warehouse to assess whether the eligibility criteria as described would yield a reasonable cohort of patients as potential subjects (protocol feasibility). These medication-related data elements then become information requirements that a Medication Profile should ideally meet, in order to be able to support these two uses in the clinical research domain.

## 1. Introduction

As part of a larger requirements gathering exercise for the in-depth analysis of the content needed in an ideal Medication Profile from both direct to patient healthcare and clinical research, requirements from two of the processes in clinical research domain were studied. This paper focuses on the medication information requirements to support protocol feasibility and patient recruitment studies. Requirements from the other contexts will be published in due course, together with a larger set of recommendations.

The provision of accurate information about the medicines that a patient is using now and has used in the past is extremely important both for the provision of direct patient care and also for the so-called “secondary uses” of information for research. This information is often termed the patient's “Medication Profile”; however there is little if any consensus as to what a Medication Profile should contain [1–5].

Subjects are selected as suitable for recruitment into a clinical study based on eligibility criteria which are formally documented as part of the protocol for the study. Finding suitable subjects is known to be difficult [6, 7], and success in recruitment is variable [8]. Various strategies are being developed to support recruitment, including design and deployment of systems for protocol feasibility studies and subject identification and recruitment [9]. These use the content of prospective or actual eligibility criteria as queries against a patient data warehouse to retrieve either numbers of the likely to be eligible patients (for feasibility testing) or individual patients (for possible recruitment).

There has been little analysis of the content of eligibility criteria and none specifically on their medication-related content. van Spall et al. [10] undertook a systematic review of the description of exclusion criteria (only) in published randomised controlled trials; 54.1% of the trials examined had “medication-related reasons” for exclusion meaning that over half of all trials studied required at least some medication-related information for eligibility assessment. Weng et al. conducted an examination of eligibility criteria specifically for their computability to support clinical research and focused on the semantic structuring of the criteria [11], rather than on their clinical content. Ross et al. [12] conducted an analysis to characterise eligibility criteria into three categories, one of which was “a treatment or intervention on the participant,” which is presumed to include medication, and to quantify their patterns and the complexity of these patterns.

The aim of this work was therefore to analyse a set of study eligibility criteria and to specifically investigate in detail the medication-related data elements which could be used as parameters to query a patient's Medication Profile to assess their suitability for entry into a trial (patient recruitment) or to query a set of Medication Profiles in a data warehouse to assess whether the eligibility criteria as described would yield a reasonable cohort of patients as potential subjects (protocol feasibility). These medication-related data elements then become information requirements that a Medication Profile should ideally meet, in order to be able to support these two uses in the clinical research domain.

A subsidiary aim was to have some sense of the value, in terms of frequency of use, of each of these data elements, such that an assessment of their importance for the particular use case can be made: if a parameter is used in many eligibility criteria, the value of its presence in the Medication Profile is high and vice versa.

## 2. Method

The analysis studied eligibility criteria from 41 clinical studies conducted in Europe by nine different pharmaceutical companies provided to the EHR4CR project [13] specifically for use in protocol feasibility and patient recruitment studies. This set of 41 trials had been selected from the total of studies in progress at the EHR4CR Pilot Sites as being representative of clinical studies currently conducted in the domain; the selection was made by the EHR4CR EFPIA partners. There were 1112 individual eligibility criteria from these studies, although there was considerable variability in what each trial considered to be a single eligibility criterion. For some studies, a single criterion might contain a number of related parameters each of which must be satisfied, whereas in other studies each parameter was detailed as a separate criterion to be satisfied. Because the investigation was seeking a qualitative understanding of the requirements that eligibility criteria place on the Medication Profile compared to a truly quantitative measure, no attempt was made to normalise the pattern of eligibility criteria such that each described one and only one parameter; the eligibility criteria have been used and counted exactly as they were supplied by their authors.

Each of the eligibility criteria was examined and those whose parameter(s) involved medication information were identified for further detailed study, including categorisation. Medication Profile information has two main parts: the identification of the medication(s) themselves and information about their use in the patient, the dosage instructions; both of these aspects were examined in more detail to elicit requirements. Eligibility criteria describing adverse events to medication occurring during the study (i.e., after a subject has been recruited into the trial) were not included for detailed evaluation (e.g., “any other hemorrhage/bleeding event > CTCAE Grade 3 within 4 weeks of first dose of study drug”). However, eligibility criteria that described adverse events to medication that had occurred prior to the study and which therefore might be expected to be documented in a patient's Medication Profile were identified and evaluated.

To provide some comparison to the medication information based data elements, eligibility criteria whose data element(s) involved laboratory test information were also identified. Laboratory test information was defined as biochemistry and haematology tests only; observables such as blood pressure measurement, pathology, and microbiology information were not included. Laboratory test information was chosen as a comparator for medication information as like medication information, it is highly structured with little additional free text and is similarly stored in patient records.

## 3. Results

Of the 1112 eligibility criteria, there were 201 that made a direct reference to medication that a potential subject may be taking or may have taken.

In addition, there were 79 eligibility criteria that described medication-related information (allergy to medication and adverse events from medication administration) that should be available from an extended Medication Profile or that could be queried by inference.

The number of eligibility criteria that involved laboratory test information was 99; 39 were inclusion criteria and 60 were exclusion criteria.

## 4. Categorisation

**Figure 1:**
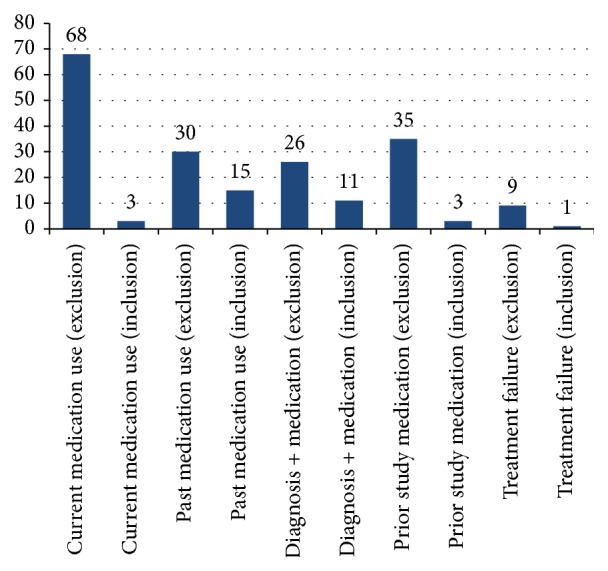
Graph showing the initial categorisation of medication-based eligibility criteria.

The 201 eligibility criteria containing reference to medication-related data elements were examined in detail and categorised on the basis of whether they referred to current or past medication use and whether they referred to medication information use in context of a diagnosis, to use of medication that was a study agent in a previous study, or to a medication failure (Figure 1).

“*Current medication use*” is defined as those eligibility criteria that identify a medication (or group of medications which might be defined by therapeutic category or by shared indication) being taken by a patient/potential subject at the time of recruitment into a study using words and were identified by phrases such as “(current) use of” or “concomitant administration of.” The large majority of these were exclusion criteria with just 2 being inclusion criteria.

Example:

 “*Currently on any medication to treat high blood pressure.*”

Included in the “current medication use” category are those eligibility criteria where there was some indication of the timing of the medication administration in relation to the current point in time or to a particular point in the study such as randomisation, for which it is likely that the medication would (still) be used “in the present.”

Example:

 “*Treatment with oral neuroleptics within 4 weeks prior to the screening visit.*”

“*Past medication use*” is the category of eligibility criteria that describe a medication (or group of medications) that has been taken in the past and is no longer being taken (words and phrases such as “prior administration or” or “history of”). Some eligibility criteria were not explicit in their reference to past medication use, for example, the criterion “at least one but not more than 2 cytotoxic chemotherapy regimens for metastatic castration-resistant prostate cancer”; this implies that the chemotherapy must have happened previously to “now” and would therefore be recorded as “past.” The “past medication use” criteria were split with two-thirds being exclusion criteria and one-third being inclusion criteria.

Example:

 “*History of prior exposure to carisbamate.*”

The set of eligibility criteria described as “*Diagnosis + Medication*” are those where the primary criterion is that the subject has the condition/symptom described (a “diagnosis”) with a supplementary qualification identifying a medication. The subject must have both the condition and symptom (diagnosis) and be using or have used the treating medication to fulfil the criterion.

Example:

 “*Cardiac arrhythmias requiring anti-arrhythmic therapy.*”

“*Prior study medication*” is the category of eligibility criteria which describe the use by a patient of a study agent in a previous study; the overwhelming majority (35) was exclusion criteria, but there were 3 that were inclusion criteria (such that the patient would be eligible as a subject for a “follow-on trial”).

Example:

 “*Investigational drug therapy outside of this trial during or within 4 weeks of study entry.*”

**Figure 2:**
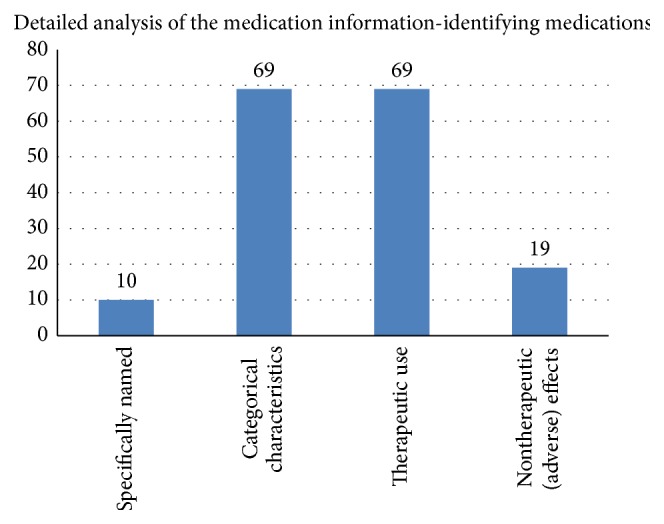
Graph of the various ways to identify medications in eligibility criteria.

There were 10 eligibility criteria that concerned “*treatment failure*” which was recognised by phrases such as “inadequate response to,” “resistance/resistant to,” and “relapse after”; with just one exception these were all inclusion criteria (Figure 2).

Example:

 “*History of inadequate response to at least 1 AED *….”(where AED = anti-epileptic drug).

Only 10 of the 201 eligibility criteria named a specific medication; all the others used a class description referring to characteristics or use of the medicines in that class.

Medications were described on the basis of their* categorical characteristics*, for example whether they are in the chemical group of “bisphosphonates” or “anthracyclines” or share a common mechanism of action, for example “beta-blockers” or “glucocorticosteroids.” These grouping features are part of the categorical information about medicines; information is always and necessarily true for that medicine.

Example:

 “*Chronic treatment with a non-steroidal anti-inflammatory drug.*”

Medications were also described and identified as a group on the basis of their* therapeutic use,* for example, whether they are used to treat hypertension or in cancer chemotherapy. This information is not part of categorical medicinal product information, it is contextual, and it changes over time. For example, for many years aspirin (acetylsalicylic acid) was used only as an analgesic and antipyretic and then it was discovered to have antithrombotic properties and so is now additionally used as secondary prevention therapy after myocardial infarction, stroke, and a variety of other cardiovascular events, so its therapeutic use has changed over time.

Example:

 “*Patients receiving antipsychotics who are not on stable doses of atypical antipsychotics for four weeks prior to baseline.*”

Just as medications can be described and identified as a group according to their therapeutic use, they can also be grouped by particularly* significant nontherapeutic effects*; these are usually considered to be unwanted and undesirable and are therefore often known as “adverse effects.” This information is also contextual and changes over time, especially as experience with the use of the medications grows.

Example:

 “*Any concomitant medication known to prolong the QT interval.*”

**Figure 3:**
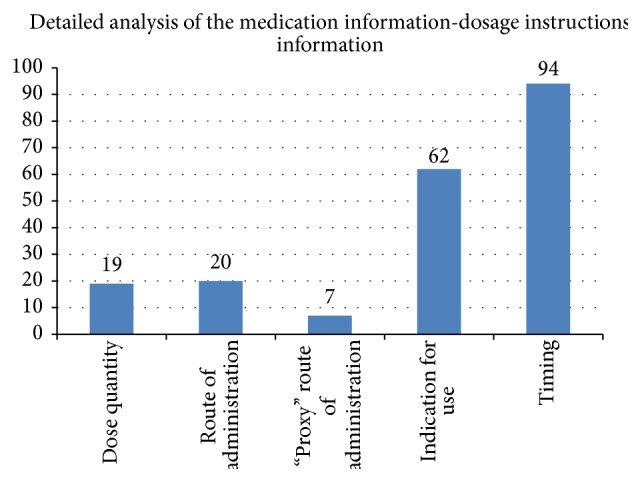
Graph of the types of dosage information in eligibility criteria.

Medications in eligibility criteria were described and identified as a group by their* authorisation status*, whether or not they have a formal marketing authorisation. This information is factual information about medicinal products at a point in time within their overall development lifecycle (Figure 3).

Example:

 “*Treatment with a non-approved or investigational drug within 30 days before Day 1 of study treatment.*”

Dosage instructions information [14] is information about dose quantity (individual dose quantity, daily dose quantity, or cumulative (“lifetime”) dose quantity), about timing of administration (frequency of individual administrations or duration of the course of therapy or start/stop dates) and about route of administration. Indication information is not considered a core component of the dosage instructions information but is assessed with them as provision of the reason for the medication can be given as part of the instructions.

Less than 10% of the medication focused eligibility criteria made any reference to* dose quantity information* and of those half of those criteria did not specify a dose quantity per se but specified “stable dosage” or “changing dosage” as part of their description, implying that dose quantity would need to be queried.

Example:

 “*Low dose warfarin (1 mg po qd) is permitted if the INR (international normalized ratio) is <1.5. Low dose aspirin is permitted (≤100 mg daily).*”

A similarly small percentage of the eligibility criteria specifically referenced one or more* routes of administration* for medications directly (oral and intravenous) and further 7 criteria referenced route of administration by the proxy grouper concept of “systemic,” which implies oral or parenteral routes of administration (as opposed to topical routes).

Example:

 “*Patients that required any use of IV vasodilators.*”

However more than a quarter of all medication focused eligibility criteria described the* indication* for use of the medication as part of their content.

Example:

 “Patients in whom anticoagulant treatment for their index PE or DVT should be continued.”

Almost half of medication-based eligibility criteria described some element of the* timing of the medication administration*. Of these, 73 were “within” and 11 were “prior to” a certain point, usually a milestone in the study lifecycle, screening, randomisation, or first dose of study medication. The others were “for at least…before.”

Interestingly, some eligibility criteria, and particularly those describing use of another investigational agent, also stated a time period in terms of “within x days or 5 half-lives, whichever is longer.”

Example:

 “*History of felbamate treatment within the past 3 months.*”

## 5. Other Types of Medication-Related Information

**Figure 4:**
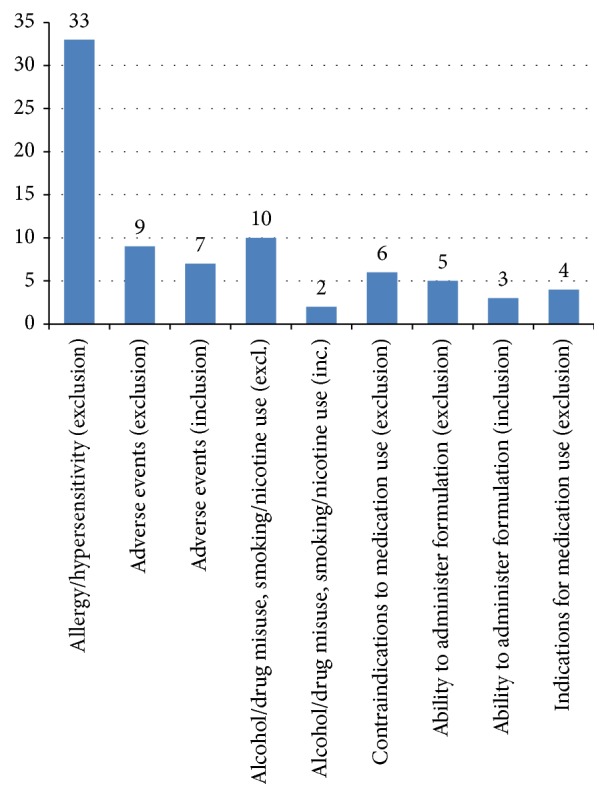
Graph of the types of medication-related eligibility criteria.

The 79 eligibility criteria that referenced medication-related information that could possibly be included in a broad Medication Profile were categorised as described in Figure 4.

All the eligibility criteria that described* allergy or hypersensitivity* to a medication or an excipient were exclusion criteria.

Example:

 “*History of hypersensitivity to docetaxel or polysorbate 80.*”

Eligibility criteria describing* adverse events* to a medication or type of medication were mostly exclusion criteria. Note that an allergy could be considered an adverse event and also included in this category but in this analysis was classified separately if specifically described (as above).

Example:

 “*Unresolved or unstable serious adverse events from prior adjuvant chemotherapy or radiotherapy.*”

There were 12 eligibility criteria looking at current or past* substance and alcohol misuse*, either directly or by referencing screening and/or current or past smoking status and nicotine use. Two criteria were listed as inclusion criteria, but they specified a “negative result” which semantically means that they were effectively exclusion criteria.

Example:

 “*Current alcohol dependence or drug abuse.*”

All the eligibility criteria which were described in terms of* contraindications* to a medication or group of medications were exclusion criteria.

Example:

 “*Contraindications to the use of corticosteroid treatment.*”

Some eligibility criteria described the* subject's ability or otherwise to administer*/have administered the medication in a particular formulation.

Example:

 “*Patients unable to swallow oral medications.*”

All the eligibility criteria which described the subject being* indicated for treatment* with a medication or group of medications were exclusion criteria; in these criteria the indication for medication use is used as a proxy for a set of diagnoses.

Example:

 “*Indication for anticoagulant therapy for a *condition other than atrial fibrillation (e.g., VTE)” (where VTE is venous thromboembolism).

## 6. Discussion

### 6.1. Importance of Medication Profile Information

The results of the analysis show that just over 18% of the 1112 examined eligibility criteria from the 41 clinical trials made reference to medication information. This information therefore needs to be present in a potential subject's Medication Profile in order to ascertain whether that person would be eligible for a study. This proportion is approximately double the proportion of examined eligibility criteria that referred to laboratory test information, which provides good evidence of the value of using a patient's Medication Profile for protocol feasibility studies and in the development of computer platforms and tools to support patient recruitment.

This proportion is considerably lower than an analysis conducted by Ross et al. [15], which found, of 1000 eligibility criteria studied, “criteria specifying treatments or interventions participant has received or will receive” accounted for 34% of the total; however no clear definition of the difference between a “treatment” and an “intervention” is provided in that analysis. The proportion of eligibility criteria referencing laboratory data was also lower in the Ross analysis, 9% as opposed to 23%; this suggests that the categorisation in this analysis was more granular than that of Ross et al.

### 6.2. Current Medication and Past Medication

The analysis shows the relatively high level of importance of a subject's current medication as compared to the medication history, in that almost twice as many eligibility criteria referred to medication administered “now” (and including prior to and including “now”) as referred to those having been administered in the past and are no longer being administered. Note that in the context of eligibility criteria the “now” may well be a specified point in the trial process, randomisation, first administration of the investigational product, and so forth. However, if the number of eligibility criteria that focus on previous administration of a study agent, which is a type of “past medication,” is included, this would give roughly equal value to knowledge of “current” and “past” medication. This is important because in the healthcare delivery domain the current medication is deemed to be the more important, for decision support, and so forth, with a longitudinal record being of lesser importance.

### 6.3. Investigational Product Use

There are a significant number of eligibility criteria that refer to previous administration of a study agent or to the authorisation status of products that have been previously administered, which includes use of an authorised medication outside of its approved indications.

This is significant because in recent years there has been a growth in the use of study registries which, accompanied by the concentration of clinical research in a smaller number of large centres, means that the reuse of potential subjects is becoming a problem.

In order to correctly interrogate a Medication Profile for use of study agents or other nonapproved drugs, it has to be possible to identify these. There are two possibilities. One is to incorporate the use of a medicines knowledge base that has wide coverage of study agents as part of the query application (see below) that could be used to compare all the medicines in a patient's profile with their authorisation status in the country of use. Unfortunately, most medicines knowledge bases do not have full coverage of investigational agents because information about these is not widely available and putting such information, even in a limited way, has sometimes been deemed “advertising,” which is not permitted for unauthorised medicines. Even though there is a move from the regulatory agencies to increase the availability of information on investigational products and an acceptance that inclusion of basic information about an unlicensed product in a knowledge base is not “advertising,” it is likely to be some several years before such information is widely available and is useable in a computable way. Even if the information was available, the comparison of each and every medicine in a profile against an authorisation status is a considerable task, especially for protocol feasibility testing when a large volume of potential patients' information is being queried. The second possibility, therefore, is to indicate directly into the Medication Profile when a patient is taking an investigational agent. This is not, to the writer's understanding, currently done in any formal way by any medication recording system (PMR or EHR) used in direct patient care but on the evidence of this analysis would appear to be useful.

### 6.4. Identifying Medicines—Knowledge Base Requirement

Only a very small number, less than 3%, of eligibility criteria that focus on the subject's use of medicines directly describe the medicines themselves; all the rest describe medicines in groups, either by categorical characteristics or by therapeutic use, or indeed by nontherapeutic effect.

This is significant for any process that wishes to use Medication Profile information in protocol feasibility studies and/or in tooling to support patient recruitment in that it introduces a requirement for knowledge about medicines to be available for use. A medicines knowledge base, such as those produced to support medication decision support in direct to patient care, should have the categorical information and the therapeutic use information readily available and in a format that would be straightforward to process to provide the additional information to support the querying of these eligibility criteria [16]. A knowledge base of this type will use the Summary of Product Characteristics (SmPC) as one of its primary sources. In that document, which is laid out in standard sections [17], the categorical characteristics of a medicine are described in the “pharmacodynamic properties” section (section 5.1 of an SmPC) usually a direct reference to a formal characteristics classification such as the ATC [18]. Therapeutic use is similarly described in the “therapeutic indications” section (section 4.1 of an SmPC).

However, although nontherapeutic effect information of the types seen in the eligibility criteria is available for medicinal products as part of this authorisation information, it is not as organised and as accessible as the categorical characteristics or indications information nor is it standardised. For although there is a section in an SmPC labelled “undesirable effects” (section 4.8 of an SmPC) this merely lists all unwanted effects that the medication has been found to cause or suspected of causing. For newer medicines these effects are at least grouped together in categories based on the MedDRA “System Organ Class” [19] hierarchy and therefore it is possible to more easily identify those of relevance to an eligibility criteria, say, by looking at the cardiac disorders for QT interval prolongation. But this type of adverse effect information may also or alternatively be described elsewhere, as in the “special warnings and precautions for use” [20] section (section 4.4 of an SmPC).

Information about enzyme modulation caused by a medicinal product may be even more dispersed in a single SmPC. It may appear in the “posology and method of administration” section (section 4.2 of an SmPC) because it is seen as a requirement for dosage adjustment; or it may appear in the “special warnings and precautions for use” part (section 4.4 of an SmPC) as information about coadministration and it will almost certainly also appear (again) in the “interaction with other medicinal products and other forms of interaction” (section 4.5 of an SmPC). Therefore, although the raw data is usually available, even if somewhat scattered in location, this has not be processed into useful knowledge for use in practice. For example, there is no well documented and agreed set of medicines acknowledged as those which prolong the QT interval in a clinically relevant manner. An illustration of this is that the British National Formulary lists QT interval prolongation as side effect of macrolide antibiotics [21] but does not do so for quinolone antibiotics although such effects have been documented [22] and indeed are noted in SmPC [20]. The same is true for cytochrome P450 isoenzyme modulators and indeed is more complicated as there are several individual isoenzymes to consider. Knowledge bases currently take this raw data on enzyme modulation and apply it in the maintenance of their drug interaction applications, rather than provide it directly as information about the medicinal products that are CYP modulators [23]. Recognising this issue, some clinical trial protocols will document lists of medications that in their context are considered to carry these risks, for example, “any concomitant medications that may cause QTc prolongation or induce Torsades de Pointes (see the Appendix for the list of medications in Tables 1 and 2) or induce CYP3A4 function” and “concomitant use of CYP3A4 inhiibitors or inducers. See Section  5.3.2 for list of prohibited medications.” But these are not standard lists and have to be managed on a study by study basis and cannot necessarily be applied to other studies that have not provided such information.

There were a small number of eligibility criteria that described alcohol and/or substance misuse and/or nicotine use. Whilst not directly part of the Medication Profile, there is information that could contribute to this. Medications used specifically in the management of substance misuse, alcohol misuse, and nicotine replacement therapy are likely to be documented in a profile and, by using a knowledge base to identify these and then querying against that set, some assessment of a potential subject's suitability against this type of eligibility criteria could be made using information in the Medication Profile.

A knowledge base could be used to provide information to assist in querying for the small number of eligibility criteria that reference contraindications to medications, by listing these contraindications as conditions and then the patient record querying for evidence of their presence directly. However, this is a considerable amount of processing for a relatively small number of eligibility criteria; it would be more constructive to protocol feasibility studies and patient recruitment support to list the conditions themselves, rather than use a medication's contraindications as a proxy.

### 6.5. Dosage Instructions

The proportion of eligibility criteria that describe use of medicines and that also referenced dosage instruction information showed some clear patterns. Less than 10% described either dose quantity, frequency of administration, or duration of the course of therapy, and a similar proportion described route of administration. However, nearly half of all eligibility criteria that describe use of medicines also reference when the course of therapy occurred, either that it was currently in progress or how long in the past it had occurred and ceased, as already described in the classification of those eligibility criteria that reference either current medication or past medication.

Given the complexity that can easily develop in describing dosage instructions information in a machine readable way, these results indicate that there is little value to be obtained by attempting complex querying of this information within the Medication Profile for protocol feasibility studies and patient recruitment tooling. But the results show that it is important for protocol feasibility studies and patient recruitment tooling to be able to ascertain the basic timing of the course of therapy (i.e., start and stop dates), even if the detail of the dose quantity, frequency of administration, or route of administration within that course is not provided in any machine readable/queryable way.

### 6.6. Indication for Treatment and “Reason to Stop”

30% of eligibility criteria that include medicinal product use also require evaluation of the indication for the use of the medication, but this information is rarely directly recorded in a Medication Profile and therefore is presently unlikely to be directly available in a clinical data warehouse or EHR system.

The information may well be present implicitly; the patient was diagnosed with breast cancer at point X and three weeks later doxorubicin is administered; it is almost certain that the doxorubicin would be indicated for the treatment of the breast cancer. So whilst a clinician reviewing a patient with a complete health record can make that connection straightforwardly, an application querying a clinical data warehouse or EHR system would find that an extremely complex task to accomplish successfully given the current state of such systems. And even a clinician may find this type of inference difficult for those medications with a diverse set of therapeutic uses; being clear of the indication for the use of amitriptyline, whose primary use has been as an antidepressant but is now as likely to be used as an analgesic in postherpetic neuralgia or as a prophylactic against migraine, is a much more tricky task.

Note that indication information in an eligibility criterion is subtly different from the combination of a particular diagnosis with a medication qualifier, although each describes a medication and a condition/symptom being treated. The latter is somewhat easier to query for in a clinical data warehouse or EHR system; the diagnosis can be queried directly, and if found, then the qualifying medication can be investigated, again directly. Since both mechanisms achieve roughly the same ends, potential subjects with a particular diagnosis also taking a particular medication that is related to that diagnosis, wording eligibility criteria in the pattern of “diagnosis + medication qualifier” is likely to be more efficient for querying in protocol feasibility studies and patient recruitment tooling than using the “medication and indication” pattern.

A small number of eligibility criteria referred directly to treatment failure; the subject had therapy with the particular medication, but it was unsuccessful. In addition, in the analysis of those criteria which were deemed as not being directly related to the Medication Profile but referenced medication in some way, the majority concerned allergy/hypersensitivity or an adverse event. If these are considered together as “reason(s) to stop” a therapy, this amounts to significant number of the eligibility criteria. “Reason to stop” information is rarely recorded directly, but these results suggest that it would be of use if it were.

Currently, there is nothing formally available that would provide information to support a query for the very small number of eligibility criteria that are concerning a potential subject's ability to self-administer particular formulations of a study agent. This type of information is more likely to be recorded in nursing and care notes than in any other part of a patient's health record.

### 6.7. Ethical and Medicolegal Implications

This analysis of eligibility criteria requirements for the Medication Profile has focused on the medication specific data elements that the profile would need to contain in order to support the systems mediated detection of suitable subjects for protocol feasibility testing and patient recruitment. However, in order for these data to be actually used in such testing or going forward as part of a clinical trial data set, it would be necessary to comply with all the appropriate ethical and medico-legal requirements, including those of Good Clinical Practice (GCP) [24]. These include the requirement that the authorship and time stamping of all data are preserved and that all changes are made in a version controlled manner permitting full traceability and the potential for rollback to a prior version of the data, a comprehensive audit trail and a long-term commitment to data retention. Many of these GCP medicolegal requirements are identical or very similar to the medicolegal requirements for electronic health record data, such as those published in ISO 18308:2011 “Requirements for an electronic health record architecture” [25].

## 7. Conclusion and Recommendations

Information from a potential subject's Medication Profile makes a significant contribution to the overall set of queries, based on study eligibility criteria, which are used in protocol feasibility studies and patient recruitment tooling.

Information on both current medication use and past medication use was shown to be equally useful, when use of a past study agent is included as a type of past medication, which supports the requirement to have longitudinal information in a Medication Profile as well as current medication information.

In terms of the detail of what is recorded in the profile in addition to the identification of the medicinal products themselves, the most useful element is the timing of the course of therapy, when it commenced and if/when it has ceased. Other dosage information, including route of administration and dose quantity, was found to have limited use. In conjunction with the start and stop timing, direct recording of information about the indication to start a therapy and reasons for its cessation were found to be of benefit in this context. Neither is currently recorded in routine practice.

The analysis demonstrated the requirement to be able to query a Medication Profile to ascertain whether any of the medications that a patient has received is an “investigational agent” (i.e., a medication which does not possess a marketing authorisation and therefore is administered as part of formal clinical research).

It was clear that, due to the way eligibility criteria are currently written, a medicines knowledge base is required to expand some grouping concepts from the criteria into individual medication concepts such that the Medication Profile can be queried directly. In addition, it is recommended that further work be undertaken in this context to produce standard and agreed sets of medications acknowledged to cause particular nontherapeutic effects that are known to be of concern in clinical trials, most particularly those medicinal products that prolong the QT interval and those that cause CYP modulation. This would have value in the wider context of clinical decision support as well as for study design and execution.

It was interesting to note the similarities and differences between two particular patterns of eligibility criteria, the “diagnosis + medication qualifier” pattern and the “medication + indication” pattern. Given the known lack of recording of indication information, the former pattern appears to be the more useful; yet the latter pattern appears to have more extensive use in eligibility criteria. Further investigation in this area could be undertaken to further explore the similarities and differences, and if there was evidence that the one pattern was significantly more effective in protocol feasibility studies and patient recruitment tooling, this should be reflected back to those working in study design. It should be noted that optimisation of the eligibility criteria given in a study protocol would be expected to optimise recruitment of subjects to that study, so this would indeed be a useful area for further work.

This study examined just over a thousand eligibility criteria from 41 phase III studies into new medications; a similar type of investigation could be conducted on other study phases and study types. This should include observational (phase IV) studies for medications and studies into other types of medical intervention (procedures and device use) to confirm if similar patterns of information requirements exist or indeed whether these types of studies place additional requirements for information on the Medication Profile.

The results of this analysis give several recommendations, beyond the provision of requirements for the content of a Medication Profile. These recommendations are primarily aimed at authors of eligibility criteria but also to authors of medicines knowledge bases and to the providers of electronic health record systems. Medication Profile information in EHR systems should be structured and designed such that their recording of medication information and in particular the granularity of the data elements within that support all of the relevant use cases for that information: high quality direct care delivery and also secondary uses, including clinical research.

If an eligibility criterion needs to identify subjects using a medication to treat a particular condition, rather than stating the medication and indication (medicine X to treat condition Y), based on this analysis it is more useful to state a “diagnosis Y and treatment with X,” as a diagnosis and a medication are much more likely to be separately present and queryable in a health record than the medication with its indication for use.

Standardised sets of the medications that are acknowledged to cause important clinical effects such as prolongation of the QT interval and CYP modulation should be developed. These should be agreed for use in both direct to patient healthcare and clinical research and should be available for use in knowledge base systems.

In the interim, when an eligibility criterion refers to such an effect, it should provide the set of medications that the author deems to be causative, to avoid different investigators using different sets.

These conclusions and recommendations will be combined with other recommendations and requirements from the analysis of other uses and processes for patient medication information and will be used to develop the specification for a cohesive and comprehensive Medication Profile which will be published later.

## Appendix

## Results Summary Tables

See Tables 1, 2, and 3.

## Figures and Tables

**Table 1:** Results summary: eligibility criteria containing medication information.

Eligibility criteria making direct reference to medication information	201 (18.1%)	178 exclusion 33 inclusion
Current medication use	71 (35.3%)	68 exclusion 3 inclusion
Prior study medication	45 (22.4%)	30 exclusion 15 inclusion
Diagnosis with medication use qualifier	37 (18.4%)	26 exclusion 11 inclusion
Other study participation	38 (18.9%)	35 exclusion 3 inclusion
Treatment failure	10 (5.0%)	9 exclusion 1 inclusion

**Table 2:** Results summary: identifying medicines in the medication information in eligibility criteria.

Medicines specifically named	10 (5.0%)
Identified by categorical characteristics (e.g., chemical group)	69 (34.3%)
Identified by therapeutic use	69 (34.3%)
Identified by nontherapeutic effects (e.g., adverse events caused)	34 (16.9%)
Identified by authorisation status	19 (9.5%)

**Table 3:** Results summary: identifying dosage instructions information in eligibility criteria.

Dose quantity	19 (9.5%)
Route of administration or “proxy”	27 (13.4%)
Indication for use	62 (30.8%)
Timing information	94 (46.8%)
